# Susceptibility of dairy cows to subacute ruminal acidosis is reflected in both prepartum and postpartum bacteria as well as odd- and branched-chain fatty acids in feces

**DOI:** 10.1186/s40104-022-00738-8

**Published:** 2022-10-05

**Authors:** Hong Yang, Stijn Heirbaut, Xiaoping Jing, Nympha De Neve, Leen Vandaele, Jeyamalar Jeyanathan, Veerle Fievez

**Affiliations:** 1 Laboratory for Animal Nutrition and Animal Product Quality, Department of Animal Sciences and Aquatic Ecology, Faculty of Bioscience Engineering, Ghent University, Campus Coupure, building F, 1st floor, Coupure Links 653, 9000 Ghent, Belgium; 2 State Key Laboratory of Grassland and Agro-Ecosystems, International Centre for Tibetan Plateau Ecosystem Management, School of Life Sciences, Lanzhou University, Lanzhou, 730000 China; 3 Animal Sciences Unit, Flanders Research Institute for Agriculture, Fisheries and Food, Scheldeweg 68, 9090 Melle, Belgium

**Keywords:** Fecal bacterial community, Fecal odd- and branched-chain fatty acids, Inter-animal variation, Subacute ruminal acidosis

## Abstract

**Background:**

The transition period is a challenging period for high-producing dairy cattle. Cows in early lactation are considered as a group at risk of subacute ruminal acidosis (SARA). Variability in SARA susceptibility in early lactation is hypothesized to be reflected in fecal characteristics such as fecal pH, dry matter content, volatile and odd- and branched-chain fatty acids (VFA and OBCFA, respectively), as well as fecal microbiota. This was investigated with 38 periparturient dairy cows, which were classified into four groups differing in median and mean time of reticular pH below 6 as well as area under the curve of pH below 6. Furthermore, we investigated whether fecal differences were already obvious during a period prior to the SARA risk (prepartum).

**Results:**

Variation in reticular pH during a 3-week postpartum period was not associated with differences in fecal pH and VFA concentration. In the postpartum period, the copy number of fecal bacteria and methanogens of unsusceptible (UN) cows was higher than moderately susceptible (MS) or susceptible (SU) cows, while the genera *Ruminococcus* and *Prevotellacea_UCG-001* were proportionally less abundant in UN compared with SU cows. Nevertheless, only a minor reduction was observed in iso-BCFA proportions in fecal fatty acids of SU cows, particularly iso-C15:0 and iso-C16:0, compared with UN cows. Consistent with the bacterial changes postpartum, the lower abundance of *Ruminococcus* was already observed in the prepartum fecal bacterial communities of UN cows, whereas *Lachnospiraceae_UCG-001* was increased*.* Nevertheless, no differences were observed in the prepartum fecal VFA or OBCFA profiles among the groups. Prepartum fecal bacterial communities of cows were clustered into two distinct clusters with 70% of the SU cows belonging to cluster 1, in which they represented 60% of the animals.

**Conclusions:**

Inter-animal variation in postpartum SARA susceptibility was reflected in post- and prepartum fecal bacterial communities. Differences in prepartum fecal bacterial communities could alert for susceptibility to develop SARA postpartum. Our results generated knowledge on the association between fecal bacteria and SARA development which could be further explored in a prevention strategy.

**Graphical abstract:**

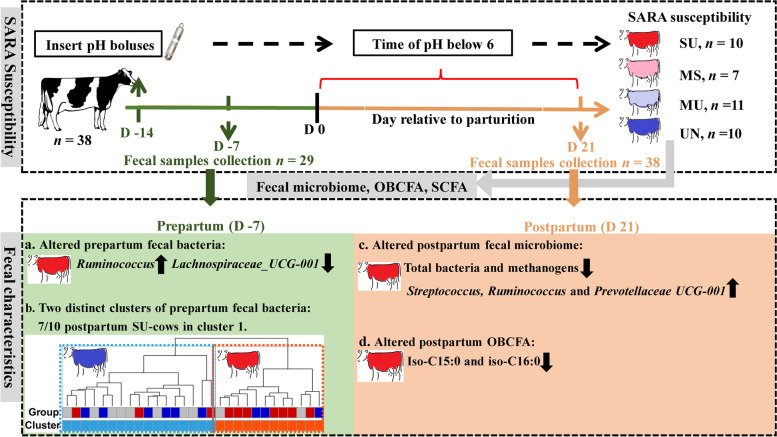

**Supplementary Information:**

The online version contains supplementary material available at 10.1186/s40104-022-00738-8.

## Background

Subacute ruminal acidosis (SARA) is characterized by episodes of ruminal pH below a certain threshold, established at 5.6 [1] or 5.8 [2] for at least 3 or 5.4 h/d, respectively. It is caused by diets with high levels of highly fermentable carbohydrates, low levels of physically effective fiber, or both [3, 4]. SARA has been reported to decrease rumen function [5–7], which could result in larger amounts of fermentable carbohydrates bypassing rumen fermentation and small intestine digestion [8]. These incompletely degraded substrates are fermented in the hindgut, which could decrease the hindgut pH and increase the risk of hindgut acidosis, the development of diarrhea, and disturbance of hindgut bacteria [1, 9–11]. These findings particularly rely on fecal observations given the difficulty of sampling large intestinal digesta [9, 12, 13]. Obviously, the ease and non-invasive character of fecal sampling could also open perspectives for monitoring SARA, with potential biomarkers including fecal pH, dry matter (DM) content, fermentation metabolites (e.g., volatile fatty acids [VFA]), and microbial profiles. Regarding the latter, both metataxonomic microbial profiling as well as indirect biomarkers such as microbial fatty acids could be considered. Indeed, fecal branched-chain fatty acid in young ruminants have been linked to diarrhea [14], while odd- and branched-chain fatty acids (OBCFA) in rumen and milk have also been associated with the incidence of SARA [15–17]. SARA development has not solely been associated with dietary characteristics because considerable inter-animal variation in SARA susceptibility has been observed [15, 18, 19]. However, it is unclear whether the inter-animal variation in SARA susceptibility is associated with variation in fecal characteristics, i.e., fecal pH, DM content, VFA and OBCFA, as well as fecal microbiota in dairy cows postpartum.

Moreover, monitoring inter-animal variability to SARA is not only of value for diagnostic purposes, but also as an early-warning tool. The observation of persistent inter-animal variation in SARA susceptibility over a long period (> 1 year) [15] is of particular interest in this respect and implies variability in SARA susceptibility might be an animal-related characteristics. This persistence could be used to identify “animals at risk” that need particular attention within the herd. Because of the build-up of concentrate in the diet, early lactation is a period of increased SARA risk [11]. As such, it would be of interest to identify animals at risk prior to the postpartum dietary challenge, e.g., during the dry period. In this regard, the hindgut bacterial community was reported to be determined by host (genetic) effects, which may be associated with the animal’s health status [20, 21] and may be resilient to dietary perturbations [22].

The objective of the current study was to investigate the association between inter-individual variation in reticular pH during the 3-week postpartum period and fecal characteristics, particularly bacterial communities, postpartum as well as prepartum. Therefore, we hypothesized that the inter-animal variation observed in SARA susceptibility is reflected in fecal characteristics in dairy cows postpartum. Furthermore, we also hypothesized that the prepartum bacterial differences allowed clustering cows with distinct postpartum SARA susceptibility. As such, we aimed to determine if fecal bacterial communities postpartum and prepartum contribute to identifying postpartum SARA susceptibility. The fecal samples of this study were obtained from an experiment with 38 dairy cows, which showed inter-animal variation in SARA susceptibility [23].

## Methods

### Animals, diets, and group assignment

All animal experimental procedures were approved by the Ethics Committee of Flanders Research Institute for Agriculture, Fisheries and Food (ILVO), Belgium (EC 2018/329). Thirty-eight Holstein-Friesian multiparous dairy cows (average age, 4.28; average lactation number, 2.99) were included in the monitoring experiment, which took place at the research farm of ILVO from 2 weeks before the predicted calving date to 3 weeks after calving in a period from March 2019 to October 2020. The cows were randomly selected from the calvings of a bigger herd (100 cows) during the 1.5 years lasting experiment. The number of animals was determined based on a SARA prevalence of 19-25% [1] under practical farming conditions (i.e., gradual build-up of grains accompanied with reduced proportions in the diet of physical structure elements).

Diets were formulated according to the Belgian-Dutch energy and protein evaluation systems: requirements and supply of protein digestible at the level of the small intestine were assessed according to the DVE system [24] and net energy requirements and supply were assessed according to the VEM system [25]. The rations were formulated following common practice on dairy farms without the attempt for SARA induction. From 3 weeks prior to predicted calving onwards, cows received a diet (688 g/kg roughage, DM basis; Table 1) based on the same partial mixed ration as the lactating cows which was further supplemented with a dry cow mineral premix (Prolacta, AVEVE, Merksem, Belgium) and on average 1 kg of balanced concentrate B (67.2 g/kg of DM) per cow per day supplied through the automatic concentrate provider (Greenfeed, C-Lock Inc., Rapid City, SD, USA). This ration was fed until 2 d after calving. The partial mixed ration of the lactating cows was calculated to fulfill the needs of an average adult cow of 650 kg, producing 26 kg of fat-protein corrected milk, and was based on maize silage, pre-wilted grass silage, pressed beet pulp, soybean meal, and balanced compound feed. The supply of balanced concentrate changed according to lactation stage and changed slightly during the course of the experiment (running over 1.5 years) in relation to the quality and feed values of the silages used. At d 3 after calving, concentrate intake at the concentrate dispenser equaled 1.7 kg of balanced concentrates (balanced compound feed A, 0.2 kg; balanced compound feed B, 1.5 kg), 0.2 kg of formaldehyde-treated soybean meal (CovaSoy, FeedValid, Poederoijen, the Netherlands) and 0.3 kg of soybean meal (Additional file 1: Tables S1). CovaSoy was increased over a period of 7 d to 1 kg, while the balanced concentrate was increased linearly to 6 kg over a period of 20 d (from d 3 to d 23). Detailed information about the ingredients, chemical composition and concentrate build-up is given in Table 1 and Additional file 1: Table S1. Cows were offered feed as two equal meals at roughly 07:30 h and 16:30 h ad libitum and had free access to water.

**Table 1:** Ingredients and chemical composition (g/kg of DM) of the diet offered from 3 weeks prior to calving (Close-up) up to the first 2 d of lactation, as well as the diets offered on d 3 and 20 in lactation (Lac3 and Lac20). A gradual linear shift from Lac3 to Lac20 took place through build-up of balanced compound feed A and B, Covasoy, and soybean meal (Table S1)

Item	Close-up	Lac3	Lac20
Maize silage	344	321	248
Grass silage	344	321	248
Beet pulp	84.2	78.4	60.7
Urea	1.40	1.30	1.01
Straw	9.57	8.92	6.90
Barley	11.0	10.2	7.89
Maize	41.6	38.8	30.0
Soybean meal	79.8	111	73.7
Balanced compound feed A^1^	—	24.5	108
Covasoy^2^	—	24.5	54.0
Balanced compound feed B^3^	67.2	61.2	162
Prolacta^4^	16.8	—	—
Chemical composition, g/kg of DM (unless noted otherwise)			
DM, g/kg	392	440	543
VEM^5^	998	1027	1053
CP	138	160	168
FOM^6^	592	591	587
Starch	168	164	180
NFC^7^	429	430	448
NDF^8^	347	334	294
ADF^9^	179	174	153
Structural value	1.82	1.71	1.37
NE_L_, MJ/kg of DM^10^	6.89	7.09	7.27
DVE^11^	73.8	103	121

^1^Contains (g/kg product): maize (430), soybean meal (270), dry beet pulp (100), wheat (85), molasses (70), feed phosphate (10), micro minerals (10), lignin-sulfonate (10), salt (6), magnesium oxide (5), and chalk (4)

^2^Covasoy = formaldehyde-treated soybean meal to bypass rumen degradation

^3^Contains (g/kg product): beet pulp (370), soybean meal (210), wheat (185), maize (120), molasses (50), salt (12), soy oil (10), feed phosphate (10), micro minerals (10), lignin-sulfonate (10), chalk (8), and magnesium oxide (5)

^4^Prolacta = commercial mineral/vitamin premix. Contains: 81.6 g of Mg, 39.5 g of P, 30.1 g of Na, 2.2 g of Ca, 0 g of K, 2500 mg of zinc sulfate, 2000 mg of choline chloride, 1250 mg of manganese oxide, 1000 mg of copper sulphate, 40 mg of sodium selenite, 20 mg of calcium iodate, 15 mg of cobalt sulfate, 1,000,000 IU of vitamin A, 200,000 IU of vitamin D_3_, and 4400 mg of vitamin E

^5^VEM = feed unit lactation [25]

^6^FOM = fermentable organic matter [24]

^7^NFC = non-fibre carbohydrates

^8^NDF = neutral detergent fibre

^9^ADF = acid detergent fibre

^10^Calculated based on the Belgian–Dutch net energy evaluation system (1000 VEM = 6.9 MJ NE_L_) [25]

^11^DVE = true protein digested in the small intestine [24]

The reticular pH of all cows was monitored every 10 to 15 min by using pH boluses (18 eBolus®, eCow, Dekon, United Kingdom; 20 SmaXtec GmbH, Graz, Austria), which omits the need for cannulated cows, which are required when using indwelling pH meters. Reticuloruminal pH boluses were inserted 17 ± 4.6 d before the expected calving date and pH was monitored up to a maximum of 3 weeks after calving. The boluses were inserted using an oral balling gun. These boluses gravimetrically end up in the reticulum, as verified by Villot et al. [26]. During the postpartum period, the 38 animals were divided into four groups based on pH criteria. For this, after complete data collection, the mean and median daily pH values were assessed against a pH threshold. A pH threshold of 6 was chosen in the current study because the reticular pH is generally 0.2 units higher than the rumen pH [27, 28]. Further, the mean and median duration that the pH dropped below 6 was calculated for each cow on a daily basis during the 3-week postpartum period. Thirty-eight cows were further ranked from high to low based on the mean and median time of pH below 6 and divided into 4 approximately even-sized groups. As such, the 38 animals were classified based on the individual cow’s mean or median time of pH below 6 or both, to allocate between 20% and 30% of the cows to each group:

- Susceptible group (SU; *n* = 10): mean or median time of postpartum pH below 6 of at least 180 min/d;
- Moderately susceptible group (MS; *n* = 7): mean time of postpartum pH below 6 < 180 min/d and median time of postpartum pH below 6 < 180 min/d;
- Moderately unsusceptible group (MU; *n* = 11): 10 min/d < mean time of postpartum pH below 6 < 60 min/d and median time of postpartum pH below 6 ≤ 30 min/d;
- Unsusceptible group (UN; *n* = 10): median time of postpartum pH below 6 = 0 min/d and mean time of postpartum pH below 6 < 10 min/d.

Because sampling in the prepartum period was based on expected calving dates, premature calving resulted in nine missing prepartum samples (samples collected from 29 prepartum cows). Of these 29 prepartum cows, ten cows were from the SU group, four cows were from the MS group, six cows were from the MU group, and nine cows were from the UN group. As such, for prepartum data analysis, data of the three cows of the MS group and the seven cows of the MU group were combined to a single moderate group (MO, *n* = 10). Three cows (SU5, severe hypocalcemia; SU7, severe lameness; SU8, hypocalcemia) in the SU group and two cows (MU2 and MU6, displaced abomasum) in the MU group showed clinical disease signs on some days during the 21-d postpartum monitoring period, whereas no signs of clinical disease were observed in the prepartum period.

### Fecal sample collection

Fecal samples were collected at a standardized timing of 2 h after the morning feeding on d 7 (± 1 d), calculated based on the expected calving date and on d 21 (± 1 d) after calving, through grab sampling (200 g) from the rectum of the cow. After homogenizing, five subsamples were immediately transferred to cryovials, snap frozen in liquid nitrogen, and stored at − 80 °C until freeze-drying for DNA extraction. The left-over fecal material was kept on ice for transport to the laboratory and stored at − 20 °C until further analyses.

### Determination of OBCFA in feces

Frozen fecal samples were thawed at 22 °C and homogenized by vigorous mixing with a spoon. A subsample of 1.5 g was freeze-dried first, followed by direct transesterification described by Vlaeminck et al. [29]. Then, the fatty acid methyl esters were analyzed by using a gas chromatograph (HP 7890A, Agilent Technologies, Diegem, Belgium) equipped with a SP-2560 capillary column (75 m × 0.18 mm inside diameter [i.d.] × 0.14 μm thickness; Supelco Analytical, Bellefonte, PA, USA) and a flame ionization detector. The column temperature of the gas chromatograph was programmed as follows: 70 °C (held for 2 min); then ramped at 15 °C/min to 150 °C; a second increase at 1 °C/min to 165 °C, a maintained for 12 min; followed by a third increase at 2 °C/min to 170 °C, maintained for 5 min; and a final increase at 5 °C/min to 215 °C, maintained for 20 min. Inlet and detector temperatures were 250 and 255 °C, respectively. Fatty acid peaks were identified by using mixtures of methyl ester standards (GLC463, NuCheck-Prep., Inc., Elysian, MN, USA). Quantification of FA was based on the area of the internal standard and on the conversion of peak areas to the weight of FA by a theoretical response factor for each FA [30, 31].

### Determination of VFA in feces

Two-hundred milligrams of frozen feces were mixed with 1 mL of distilled water and 0.1 mL of internal standard (10 mg/mL 2-ethylbutyric acid of formic acid, Sigma-Aldrich, Diegem, Belgium). After shaking for 5 min, the fluid was centrifuged at 31,000 × *g* for 15 min at 4 °C, and the supernatant was analyzed for VFA using a gas chromatograp (HP7890A; Agilent Technologies, Diegem, Belgium) equipped with a Supelco Nukol capillary column (30 m × 0.25 mm i.d. × 0.25 μm thickness; Sigma Aldrich, Diegem, Belgium) and a flame ionization detector according to the method of Dewanckele et al. [32].

### Bacterial community analysis based on 16S ribosomal RNA (rRNA) sequencing

#### DNA extraction

A total of 100 mg of freeze-dried fecal samples were homogenized and weighed before genomic DNA extraction using the repeated bead beating plus column purification (RBB + C) method [33]. The concentration and quality of the extracted DNA was checked with a NanoDrop spectrophotometer (VWR International BVBA, Leuven, Belgium).

#### Bacterial 16S rRNA gene amplicon sequencing and data mining

Extracted genomic DNA was submitted to Macrogen (Seoul, Korea) for library preparation and bacterial 16S rRNA gene amplicon sequencing (V3–V4 region, primers: 344F and 806R) [34]. Preparation of the amplicon barcoded library was based on the Illumina 16S metagenomic sequencing library preparation protocol (https://support.illumina.com). The sequencing was performed using Illumina MiSeq V3-technology (2 × 300 base pairs [bp]).

The amplicon sequencing dataset was demultiplexed and barcodes were clipped off by the sequence provider. The amplicon sequencing data were analyzed by using Quantitative Insights Into Microbial Ecology 2 (QIIME2, version 2020.08) [35]. The sequences were demultiplexed, barcodes were removed, and forward and reverse reads were imported into QIIME2. The DADA2 pipeline was used to detect and correct Illumina amplicon sequences, to remove primers and chimeric reads, and to assemble into amplicon sequence variants (ASV) [36]. A further filtering step was performed to remove low-abundance sequences with frequencies < 0.01% or present in less than two out of the 77 samples. Finally, to normalize the number of sequences per sample, a cut-off value (33,787) was chosen based on alpha rarefaction curves for all samples. Taxonomy was assigned by using a naïve Bayes classifier trained on the Silva database (SILVA Release 138, https://www.arb-silva.de/silva-license-information/) at 99% similarity followed by removal of the features of archaea and unassigned taxa [37]. Sequence files associated with each sample have been submitted to the NCBI Sequence Read Archive (SRA; https://www.ncbi.nlm.nih.gov/sra; Accession Number: PRJNA774499).

### Microbial population analysis by quantitative polymerase chain reaction (qPCR)

The abundance of the 16S rRNA gene of total bacteria, the *mcrA* gene of methanogens, the 18S rRNA gene of protozoa, and the 5.8S rRNA of anaerobic fungi (*Neocallimastigales*) were quantified by qPCR. The primers are presented in Additional file 1: Table S2. Primer sets and qPCR conditions used were as reported for general bacteria [38], fungi [39], protozoa [40], and methanogens [41]. The qPCR reactions were assayed in a 12.5-μL reaction mixture contained 6.25 μL of Maxima® SYBR Green/ROX qPCR Master Mix (2×) (Thermo Fisher Scientific, Waltham, MA, USA), 1 μL of primer mixture containing 0.5 μmol/L of each primer, DNA (20 ng), and molecular water. Amplification of each target group was carried out in a two-step cycling protocol (StepOne Real Time PCR System, Applied Biosystems, Waltham, USA) with the following program: initial denaturation at 95 °C for 10 min and 35 cycles at 95 °C for 15 s (denaturation) and 60 °C for 1 min (annealing/extension). The melting curve was built by measuring the fluorescence emissions with increased temperature from 60 to 95 °C with ramps of 0.5 °C every 15 s. Duplicate qPCR quantification was performed on 20 ng of extracted DNA. A plasmid containing a single copy of the target gene was used for qPCR standards. The copy numbers in the standards were calculated based on the DNA concentrations determined by the NanoDrop spectrophotometer (VWR International BVBA, Leuven, Belgium). External standards were prepared and used in each qPCR run to determine the gene copies in the samples. The absolute quantity of each group of microorganisms was calculated by using the respective standards and expressed as corresponding gene copies/mL of sample.

### Data analysis

The normality of the reticular pH, DM intake, fecal pH, DM content, VFA profile, OBCFA profile, and qPCR data was confirmed with quantile–quantile plots and the Shapiro–Wilk test, and the homogeneity of variances was evaluated with Levene’s test. Normally distributed data were analyzed with R (version 4.0.3) [42] using the one-way analysis of variance (ANOVA) model in the car package [43] with group as the main factor. Differences between means were determined by using Tukey’s test for multiple comparisons. Non-normally distributed data were evaluated with the Kruskal–Wallis test followed by a pairwise Wilcoxon Rank Sum test.

Bacterial sequencing profiles were analyzed in QIIME2 (version 2020.08) [35] and R (version 4.0.3) [42]. First, a nonparametric Kruskal–Wallis test was used to evaluate the differences of α diversity metrics across groups (QIIME2 software; version 2020.08) [35]. In addition, distance-based (Bray–Curtis distance) permutational multivariate analysis of variance (PERMANOVA) was carried out to check whether bacterial composition varies between groups in QIIME2 [35], subjected to unsupervised hierarchical clustering using R pheatmap package [44].

Analysis of composition of microbiomes (ANCOM) [45] tests were run in R (version 4.0.3) [42] at the phylum, family, and genus levels to determine which bacterial groups were differentially abundant among the groups of cows. The differential taxa were computed by controlling for false discoveries using Benjamini–Hochberg correction at the 5% level of significance. Further, the taxa that differed significantly across groups detected by the ANCOM test with > 0.01% relative abundance were subject to the Wilcoxon rank-sum test to assess inter-group differences.

For all tests, *P* value < 0.05 was used to define significance, with trends declared at 0.05 < *P* < 0.10.

## Results

### Dry matter intake (DMI) and reticular-ruminal pH parameters

Individual daily median and mean time of pH below 6 as well as the number of days with more than 330 min of pH below 6 during the 3-week postpartum period and during the 1-week prepartum period are presented in Tables S3. Moreover, these four groups of cows concomitantly differed from each other in postpartum daily median and mean time of pH below 6, days of time of pH below 6 > 330 min/d in 21 d and the total area under the curve of pH below 6 in the first 21 d postpartum (*P* < 0.001; Table 2). Within the first 3 weeks in lactation, groups UN and MU did not show a single day with more than 330 min of pH below 6, whereas a larger number of such days were observed in the MS and SU groups (Table 2). In line with the observations during the postpartum period, UN cows showed lower prepartum daily median (*P* = 0.006) and mean time of pH below 6 (*P* = 0.014) and a lower number of days with more than 330 min of pH below 6 > 330 min/d (*P* = 0.004) as well as a reduced total area under the curve of pH below 6 in the last 7 d prior to calving (*P* = 0.006) than SU cows. In contrast, the average daily DMI of the first 21 d postpartum and 7 d prepartum did not differ between groups (*P*_prepartum_ = 0.311, *P*_postpartum_ = 0.164).

**Table 2:** Average DMI and reticular pH characteristics in the 1-week prepartum period (based on real calving dates) as well as the 3-week postpartum period in relation to variation in SARA susceptibility over the first 3 weeks postpartum^1^

Item	Prepartum			SEM^4^	*P-*value	Postpartum				SEM^4^	*P-*value
	SU	MO	UN			SU	MS	MU	UN		
Average DMI, kg/d	14.7	14.5	15.8	0.37	0.311	19.6	20.0	19.8	21.9	0.43	0.164
Reticular pH											
Median time of pH below 6, min/d	193^a^	13.3^a^	0.00^b^	31.870	0.006	321^a^	46.4^b^	10.0^c^	0.0^d^	26.75	< 0.001
Mean time of pH below 6, min/d	182^a^	8.00^ab^	0.00^b^	31.933	0.014	356^a^	104^b^	32.5^c^	2.78^d^	26.963	< 0.001
Days with time of pH below 6 > 330 min/d^2^	1.30^a^	0.00^b^	0.00^b^	0.256	0.004	8.82^a^	1.57^b^	0.00^c^	0.00^c^	0.775	< 0.001
Mean daily area under curve pH below 6^3^	185.9^a^	8.44^a^	0.00^b^	36.476	0.006	882^a^	293^b^	50.1^c^	3.85^d^	77.548	< 0.001

^1^Postpartum grouping: susceptible group (SU; *n* = 10): mean or median of time below pH 6 of at least 180 min/d; moderately susceptible group (MS; *n* = 7): 60 min/d < mean time of pH below 6 < 180 min/d and median time of pH below 6 < 180 min/d; moderately unsusceptible group (MU; *n* = 11): 10 min/d < mean time of pH below 6 < 60 min/d and median time of pH below 6 ≤ 30 min/d; unsusceptible group (UN; *n* = 10): median time pH below 6 = 0 min/d and mean time pH below 6 < 10 min/d. Due to early calving, 10 cows were from the SU group, the UN-group only contained 9 animals in the prepartum period and MS (*n* = 3) and MU (*n* = 7) cows were merged into a single group (MO, *n* = 10)

^2^Days with time of pH below 6 > 330 min/d during 7 d prepartum or 21 d postpartum

^3^Total area under the curve pH below 6.0 (pH × min) during 7 d prepartum or 21 d postpartum

^4^SEM = standard error of the mean

^a,b^Means within a row with different superscripts differ significantly (*P* ≤ 0.05)

### Fecal microbial populations of cows analyzed by qPCR

**Fig. 1:**
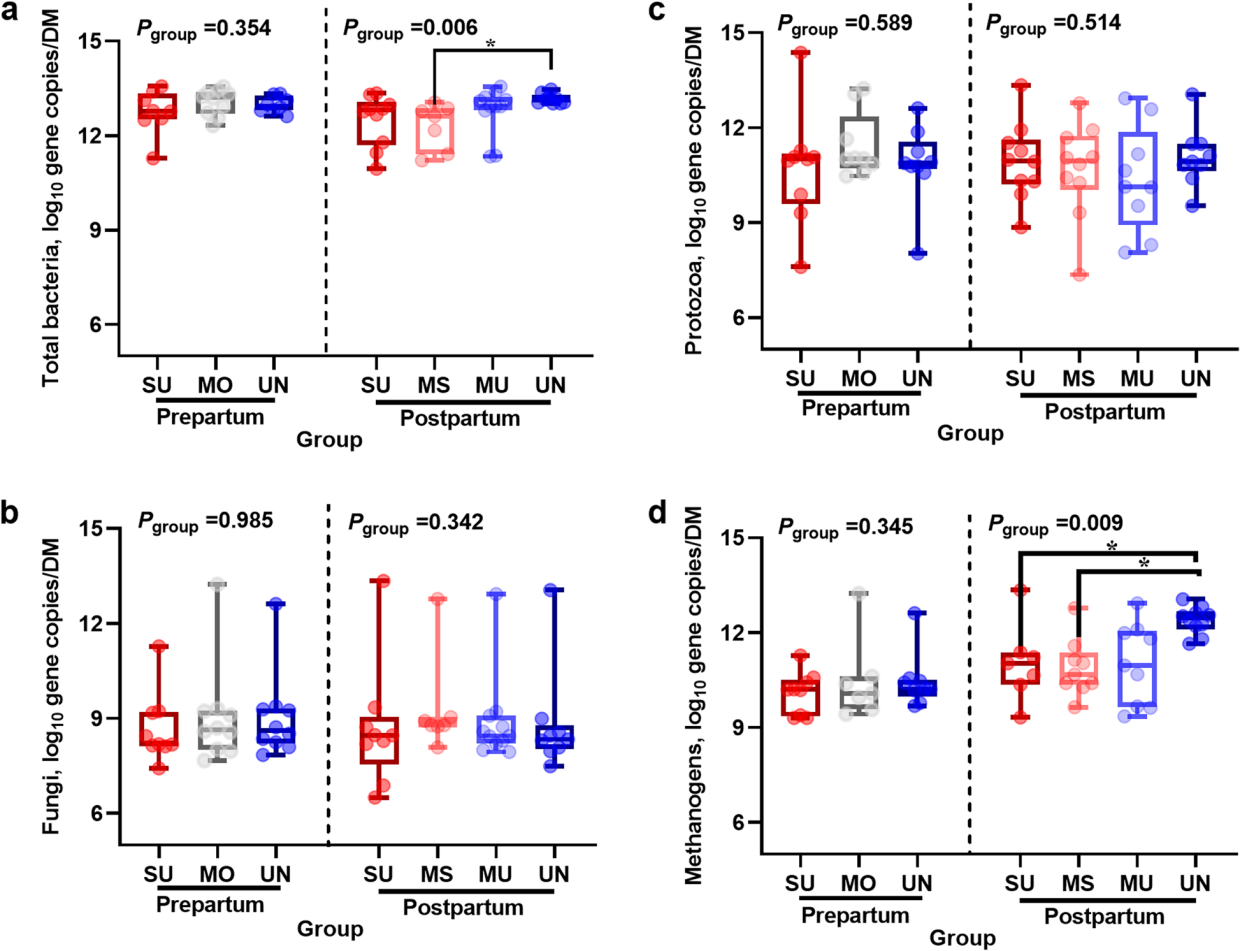
Gene copy numbers of fecal microbiota analysed by quantitative PCR in samples collected 7 d prepartum (relative to the expected calving date) as well as 21 d postpartum. Groups differed in SARA susceptibility during the first 3 postpartum weeks. *Mutually different means *P* ≤ 0.05. Postpartum grouping: susceptible group (SU; *n* = 10): mean or median of time below pH 6 of at least 180 min/d; moderately susceptible group (MS; *n* = 7): 60 min/d < mean time of pH below 6 < 180 min/d and median time of pH below 6 < 180 min/d; moderately unsusceptible group (MU; *n* = 11): 10 min/d < mean time of pH below 6 < 60 min/d and median time of pH below 6 ≤ 30 min/d; unsusceptible group (UN; *n* = 10): median time pH below 6 = 0 min/d and mean time pH below 6 < 10 min/d. Due to early calving, the prepartum groups included 10 cows from the SU group, the UN-group only contained nine animals and the MS (*n* = 3) and MU (*n* = 7) cows were merged into a single group (MO, *n* = 10)

Gene copy numbers of fecal bacteria, fungi, protozoa, and methanogens assessed by qPCR are shown in Fig. 1. The 16S rRNA gene copy numbers of bacteria and methanogens were greater in the UN cows than in the SU and/or MS cows in the postpartum period (*P*_bacteria_ = 0.006; *P*_methanogens_ = 0.009), whereas there were no differences in the prepartum period (*P* > 0.05). In contrast, the groups did not differ in protozoal and fungal gene copy numbers in the prepartum or postpartum periods.

### Fecal bacterial community composition

**Fig. 2:**
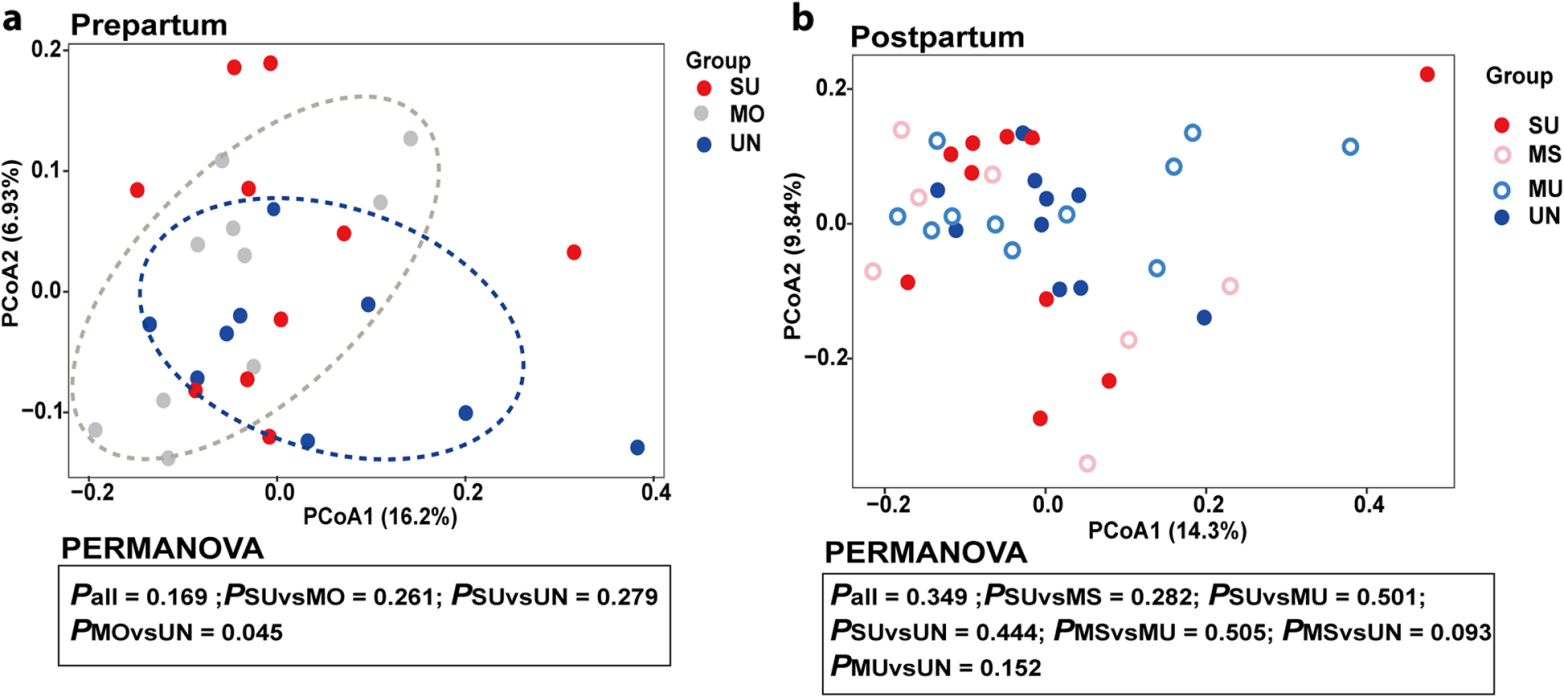
Principal-coordinate analysis (PCoA) based on Bray–Curtis dissimilarities in the composition of fecal bacterial communities at the amplicon sequence variant (ASV) level sampled on d 7 prepartum (**a**) and d 21 postpartum (**b**). Individual points in each plot represent the bacteria communities at the ASV level of an individual dairy cow and groups are indicated according to post-partum SARA susceptibility. Percentages shown along the axes represent the proportion of dissimilarities captured by PCoA in the two-dimensional (2D) coordinate space. *P* values of the permutational analysis of variance (PERMANOVA) model assessing differences in beta diversity > 0.1 are marked in the figure. Postpartum grouping: susceptible group (SU; *n* = 10): mean or median of time below pH 6 of at least 180 min/d; moderately susceptible group (MS; *n* = 7): 60 min/d < mean time of pH below 6 < 180 min/d and median time of pH below 6 < 180 min/d; moderately unsusceptible group (MU; *n* = 11): 10 min/d < mean time of pH below 6 < 60 min/d and median time of pH below 6 ≤ 30 min/d; unsusceptible group (UN; *n* = 10): median time pH below 6 = 0 min/d and mean time pH below 6 < 10 min/d. Due to early calving, the UN-group only contained nine animals in the prepartum period and MS (*n* = 3) and MU (*n* = 7) cows were merged into a single group (MO, *n* = 10)

In the postpartum period, alpha diversity indices of the fecal bacterial community (observed ASV, Faith_pd, evenness index, and Shannon index) did not differ (*P* > 0.05) among the groups (Table 3), whereas in the prepartum period the evenness of the fecal bacterial community tended to differ among the groups (*P* = 0.066). Considering beta diversity, the Bray–Curtis distance of UN cows differed from MO cows in the prepartum period (*P* = 0.042; Fig. 2a). In agreement with the difference in the prepartum period, PERMANOVA analysis revealed UN cows tended to differ from MS cows in the postpartum period (*P* = 0.093; Fig. 2b).

**Table 3:** Alpha diversity indices of the fecal bacterial community sampled prepartum d 7 and on postpartum d 21 in relation to variation in SARA susceptibility over the first 3 postpartum weeks^1^

Item	Prepartum			SEM^2^	*P-*value	Postpartum				SEM^2^	*P-*value
	SU	MO	UN			SU	MS	MU	UN		
Observed ASV	905	938	895	16.7	0.566	810	765	736	764	25.9	0.699
Faith_pd	52.2	53.6	51.6	0.78	0.585	48.3	44.4	47.5	47.0	1.13	0.480
Pielou Evenness	0.924	0.928	0.915	0.0023	0.066	0.911	0.903	0.903	0.902	0.0043	0.796
Shannon Index	9.07	9.16	8.97	0.044	0.219	8.55	8.62	8.57	8.63	0.095	0.721

^1^Postpartum grouping: susceptible group (SU; *n* = 10): mean or median of time below pH 6 at least 180 min/d; moderately susceptible group (MS; *n* = 7): 60 min/d < mean time of pH below 6 < 180 min/d and median time of pH below 6 < 180 min/d; moderately unsusceptible group (MU; *n* = 11): 10 min/d < mean time of pH below 6 < 60 min/d and median time of pH below 6 ≤ 30 min/d; unsusceptible group (UN; *n* = 10): median time pH below 6 = 0 min/d and mean time pH below 6 < 10 min/d. Due to early calving, 10 cows were from the SU group, the UN-group only contained 9 animals in the prepartum period and MS (*n* = 3) and MU (*n* = 7) cows were merged into a single group (MO, *n* = 10)

^2^SEM = standard error of the mean

^a,b^Means within a row with different superscripts differ significantly (*P* ≤ 0.05)

Table 4 shows the relative abundances of bacterial families and genera in feces sampled at d 21 postpartum which differed among cows varying in SARA susceptibility. Phyla with relative abundance higher than 1% are also reported in Table 4, although no inter-group differences were observed at the phylum level. Within the two most abundant phyla, namely Firmicutes (74.0% ± 2.44%) and Bacteroidota (20.6% ± 3.07%), one family and six genera differed among the groups at d 21 postpartum (*P*_adj_ ≤ 0.05). The family Streptococcaceae was more abundant in the feces of SU cows compared with MU and MS cows (*P*_adj_ = 0.022). Moreover, at the genus level, the relative abundance of *Streptococcus*, *Ruminococcus*, *Anaerosporobacter*, *Candidatus_Stoquefichus*, and *Prevotellacea_UCG-001* were higher in SU cows than in UN and in some cases in MS and MU cows (*P*_adj_ ≤ 0.05).

**Table 4:** Average relative abundance (%) of bacterial groups in feces sampled on d 21 postpartum that differ among groups of dairy cows varying in SARA susceptibility over the first 3 postpartum weeks^1^

Phylum	Family	Genus	SU^2^	MS^2^	MU^2^	UN^2^	SEM^3^	*P*_adj_^4^
Firmicutes			71.6	76.7	72.3	75.4	1.27	0.127
	Streptococcaceae		0.035^a^	0.002^b^	0.010^b^	0.022^b^	0.0032	0.022
		*Streptococcus*	0.035^a^	0.002^b^	0.010^b^	0.018^b^	0.0029	< 0.001
	Ruminococcaceae	*Ruminococcus*	2.14^a^	2.33^a^	2.15^a^	1.53^b^	0.154	0.008
		*Anaerosporobacter*	0.037^a^	0.018^a^	0.027^a^	0.000^b^	0.0082	< 0.001
	Erysipelatoclostridiaceae	*Candidatus_Stoquefichus*	0.313^a^	0.103^b^	0.198^b^	0.231^a^	0.0431	< 0.001
	Clostridia_UCG-014	*Clostridia_UCG-014*	1.45^b^	2.83^a^	1.34^b^	1.34^b^	0.143	< 0.001
Bacteroidota			23.2	16.5	22.7	20.0	1.37	0.338
	Prevotellaceae	*UCG-001*	0.516^a^	0.300^ab^	0.421^ab^	0.279^b^	0.0403	< 0.001
Proteobacteria			0.466	1.13	1.29	0.592	0.1812	0.971
Actinobacteria			1.47	1.42	1.22	0.74	0.261	0.971
Patescibacteria			1.43	2.09	1.23	1.74	0.188	0.606
Spirochaetota			0.859	1.06	0.552	0.856	0.1216	0.971

^1^Only differentially abundant taxa with relative abundance ≥0.01% are included here. Phyla with a relative abundance ≥1% are also presented here, even if they did not differ among groups

^2^Postpartum grouping: susceptible group (SU; *n* = 10): mean or median of time below pH 6 of at least 180 min/d; moderately susceptible group (MS; *n* = 7): 60 min/d < mean time of pH below 6 < 180 min/d and median time of pH below 6 < 180 min/d; moderately unsusceptible group (MU; *n* = 11): 10 min/d < mean time of pH below 6 < 60 min/d and median time of pH below 6 ≤ 30 min/d; unsusceptible group (UN; *n* = 10): median time pH below 6 = 0 min/d and mean time pH below 6 < 10 min/d. Due to early calving, 10 cows were from the SU group, the UN-group only contained nine animals in the prepartum period and MS (*n* = 3) and MU (*n* = 7) cows were merged into a single group (MO, *n* = 10)

^3^SEM = standard error of the mean

^4^*P*_adj_ = *P* value adjusted for false discovery rate at 5%

^a,b^Means within a row with different superscripts differ significantly (*P* ≤ 0.05)

In the prepartum period, four phyla with a relative abundance > 1% were identified in 29 samples, with Firmicutes (65.7% ± 3.57%) representing the dominant phylum (Table 5). The higher relative abundance of the genus *Ruminococcus* in the feces of SU cows 1-week prepartum (*P*_adj_ = 0.024) was in line with the postpartum day 21 observation. In addition, *Lachnospiraceae_UCG-001* were more abundant in UN cows than SU cows in the prepartum period (*P*_adj_ = 0.009).

**Table 5:** Average relative abundance (%) of bacterial groups in feces sampled on d 7 prepartum that differ among groups of dairy cows varying in SARA susceptibility over the first 3 postpartum weeks^1^

Phylum	Family	Genus	SU^2^	MO^2^	UN^2^	SEM^3^	*P*_adj_^4^
Bacteroidota			30.5	26.7	29.1	1.19	0.227
	p-2534-18B5_ gut_group	*p-2534-18B5_ gut_group*	0.447^a^	0.233^b^	0.553^a^	0.0751	< 0.001
Firmicutes			64.3	66.7	66.0	1.07	0.385
	Lachnospiraceae	*UCG-001*	0.185^b^	0.181^b^	0.335^a^	0.0234	0.009
	Ruminococcaceae	*Ruminococcus*	0.925^b^	1.30^a^	0.781^c^	0.0222	0.024
Verrucomicrobia			1.42	1.31	0.700	0.1355	0.121
Spirochaetota			1.61	2.51	1.54	0.193	0.542

^1^Only differentially abundant taxa with relative abundance > 0.01% are included here. Phyla with a relative abundance ≥1% are also presented here, even if they did not differ among groups

^2^Postpartum grouping: susceptible group (SU; *n* = 10): mean or median of time below pH 6 of at least 180 min/d; moderately susceptible group (MS; *n* = 7): 60 min/d < mean time of pH below 6 < 180 min/d and median time of pH below 6 < 180 min/d; moderately unsusceptible group (MU; *n* = 11): 10 min/d < mean time of pH below 6 < 60 min/d and median time of pH below 6 ≤ 30 min/d; unsusceptible group (UN; *n* = 10): median time pH below 6 = 0 min/d and mean time pH below 6 < 10 min/d. Due to early calving, 10 cows were from the SU group, the UN-group only contained nine animals in the prepartum period and MS (*n* = 3) and MU (*n* = 7) cows were merged into a single group (MO, *n* = 10)

^3^SEM = standard error of the mean

^4^*P*_adj_ = *P* value adjusted for false discovery rate at 5%

^a,b^Means within a row with different superscripts differ significantly (*P* ≤ 0.05)

### Prepartum fecal bacterial community and the prepartum to postpartum shift

**Fig. 3:**
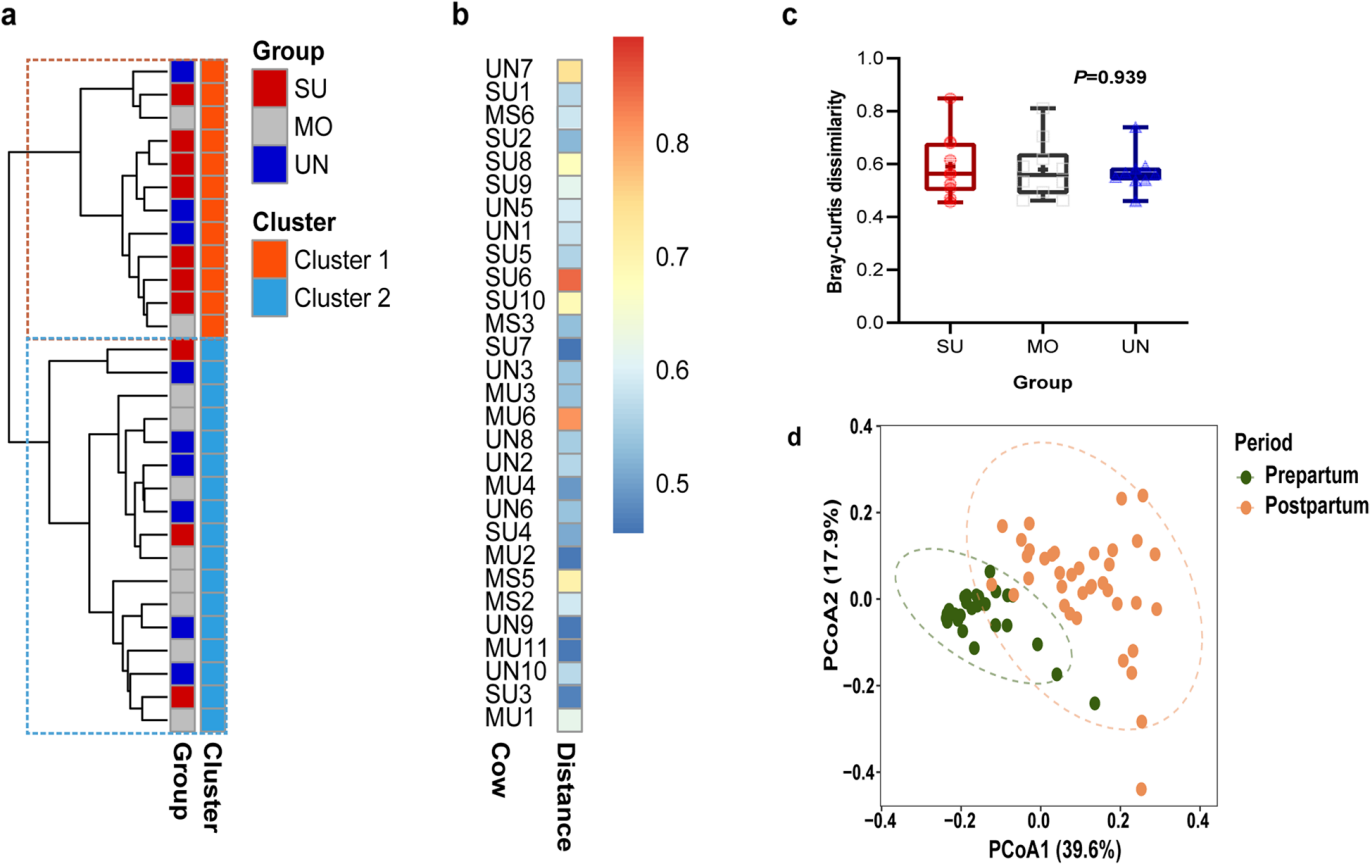
Clustering of the prepartum fecal bacterial community of cows with distinct SARA susceptibility based on Bray–Curtis distance (**a**); heatmap of the fecal bacterial community indicating the Bray–Curtis distance between prepartum and postpartum fecal bacterial communities within the same cow (**b**); comparison of Bray–Curtis distance observed in Fig. 3b among susceptible (SU), moderate (MO), and unsusceptible (UN) groups (**c**); principal-coordinate analysis (PCoA) based on Bray–Curtis dissimilarities in the composition of fecal bacterial communities at the amplicon sequence variant (ASV) level sampled either at prepartum d 7 or postpartum d 21 (**d**). Postpartum grouping: susceptible group (SU; *n* = 10): mean or median of time below pH 6 of at least 180 min/d; moderately susceptible group (MS; *n* = 7): 60 min/d < mean time of pH below 6 < 180 min/d and median time of pH below 6 < 180 min/d; moderately unsusceptible group (MU; *n* = 11): 10 min/d < mean time of pH below 6 < 60 min/d and median time of pH below 6 ≤ 30 min/d; unsusceptible group (UN; *n* = 10): median time pH below 6 = 0 min/d and mean time pH below 6 < 10 min/d. Due to early calving, the UN-group only contained nine animals in the prepartum period and MS (*n* = 3) and MU (*n* = 7) cows were merged into a single group (MO, *n* = 10)

An unsupervised hierarchical cluster analysis based on Bray–Curtis similarities was performed to test the possibility that the prepartum bacterial community clusters cows with different postpartum SARA susceptibility. Prepartum fecal bacterial community clustering at the ASV level separated the 29 cows into two distinct clusters, in which cluster 1 predominantly included SU cows (seven SU, two MO, and three UN cows; Fig. 3a). As such, 58% of the animals in cluster 1 were SU cows, with 70% of the SU cows belonging to cluster 1. On the other hand, cluster 2 included 80% of the MO cows and 67% of the UN cows. Hence, the prepartum fecal bacterial community to some extent allowed distinguishing cows that differed in the postpartum SARA pattern. The similarity of the pre- and postpartum fecal bacterial composition for each of the 29 animals was visualized by a heatmap (Fig. 3b). On average, the prepartum and postpartum fecal communities showed only 60% similarity. The prepartum–postpartum similarities did not differ between postpartum SARA groups (*P* > 0.05; Fig. 3c). Indeed, there was greater similarity within prepartum and postpartum samples than within different SARA groups, as shown by the clear separation of samples between the postpartum and prepartum period in the principal coordinate analysis (PCoA; Fig. 3d).

### Fecal pH, DM content, VFA profile and OBCFA profile

Neither fecal pH, DM content, nor VFA profile differed among the groups either in the prepartum or postpartum period (Table 6). Overall, eight OBCFA were identified in the feces of dairy cows, including four iso-BCFA (iso-C14:0, iso-C15:0, iso-C16:0, and iso-C17:0), two anteiso-BCFA (anteiso-C15:0 and anteiso-C17:0), and two odd-chain fatty acids (C15:0 and C17:0). Only iso-C15:0 differed among groups in postpartum feces. It was higher in UN and MS cows than in SU cows (*P* = 0.012). A similar trend was observed for iso-C16:0, which tended to be higher in the UN and MS groups than in the SU group (*P* = 0.099). In the prepartum period, however, no differences were observed in OBCFA among the groups (*P* > 0.05).

**Table 6:** Prepartum and postpartum fecal pH, dry matter content, volatile fatty acid (VFA) concentrations, and individual proportions as well as odd- and branched-chain fatty acid (OBCFA) concentrations of cows varying in SARA susceptibility over the first 3 postpartum weeks^1^

Item	Prepartum			SEM^2^	*P-*value	Postpartum				SEM^2^	*P-*value
	SU	MO	UN			SU	MS	MU	UN		
Fecal pH	6.89	6.90	6.86	0.042	0.934	6.47	6.57	6.38	6.41	0.032	0.252
Dry matter, g/kg	144	145	134	3.7	0.227	147	129	138	125	4.0	0.213
Total VFA, mmol/L	57.6	58.1	52.1	1.63	0.196	70.1	67.4	69.4	63.0	1.51	0.458
% of Total VFA											
Acetate	75.0	75.2	75.5	0.23	0.614	75.1	74.3	75.2	74.6	0.19	0.717
Propionate	15.0	14.9	15.0	0.20	0.968	14.6	14.9	14.7	15.3	0.14	0.202
Butyrate	6.98	7.02	6.65	0.046	0.503	8.11	8.27	8.01	7.99	0.159	0.969
Isobutyrate	1.08	0.972	1.01	0.1574	0.607	0.768	0.822	0.593	0.653	0.0368	0.243
Isovalerate	0.821	0.81	0.657	0.0816	0.649	0.437	0.587	0.313	0.266	0.0542	0.311
Valerate	1.24	1.14	1.14	0.048	0.671	1.19	1.11	1.14	1.21	0.031	0.629
Odd- and branched-chain fatty acids, mg/100 g dry feces											
anteiso-C15:0	37.6	39.6	35.7	1.99	0.299	41.6	47.5	41.3	42.3	1.30	0.395
anteiso-C17:0	22.9	24.9	22.2	1.37	0.117	21.1	24.9	21.8	21.3	1.13	0.662
iso-C14:0	9.12	10.1	9.90	0.473	0.392	8.80	10.1	9.16	9.60	0.252	0.378
iso-C15:0	16.5	17.6	15.9	0.86	0.343	15.0^b^	19.6^a^	16.7^ab^	18.8^a^	0.55	0.012
iso-C16:0	15.2	17.5	15.1	0.90	0.158	12.5	15.6	14.3	15.0	0.60	0.099
Iso-C17:0	28.6	29.1	25.7	2.01	0.469	29.1	33.2	28.8	29.2	1.53	0.778
C15:0	53.1	61.4	53.5	3.27	0.177	56.5	63.2	57.3	58.1	2.73	0.862
C17:0	60.0	63.6	58.8	2.66	0.524	56.1	60.2	55.1	53.9	2.79	0.905
Total odd-chain FA	113	125	112	6.03	0.284	112	123	112	112	5.4	0.264
Total BCFA	130	139	125	7.2	0.163	128	151	132	136	4.6	0.524
Total iso-BCFA	69.4	74.3	66.6	3.98	0.129	65.4	78.5	69.0	72.6	2.57	0.565
Total anteiso-BCFA	60.5	64.5	57.9	3.30	0.104	62.7	72.4	63.1	63.7	2.29	0.498
Total odd BCFA	106	113	99.5	5.8	0.485	108	125	109	112	4.0	0.534
Total even BCFA	24.2	27.7	25.0	1.34	0.123	21.3	25.7	23.5	24.6	0.81	0.556

^1^Postpartum grouping: susceptible group (SU; *n* = 10): mean or median of time below pH 6 of at least 180 min/d; moderately susceptible group (MS; *n* = 7): 60 min/d < mean time of pH below 6 < 180 min/d and median time of pH below 6 < 180 min/d; moderately unsusceptible group (MU; *n* = 11): 10 min/d < mean time of pH below 6 < 60 min/d and median time of pH below 6 ≤ 30 min/d; unsusceptible group (UN; *n* = 10): median time pH below 6 = 0 min/d and mean time pH below 6 < 10 min/d. Due to early calving, 10 cows were from the SU group, the UN-group only contained nine animals in the prepartum period and MS (*n* = 3) and MU (*n* = 7) cows were merged into a single group (MO, *n* = 10)

^2^SEM = standard error of the mean

^a,b^Means within a row with different superscripts differ significantly (*P* ≤ 0.05)

## Discussion

Inter-animal variation in reticular pH was observed over the 3-week postpartum period, which allowed dividing cows into four groups based on their postpartum reticular pH (i.e., SU, MS, MU, and UN cows; Table S3). All susceptible cows experienced SARA (defined as reticular pH below 6 for more than 330 min/d [2]) during at least 2 d of the 3-week postpartum period. We hypothesized these rumen pH differences could result in a distinct bacterial pattern in the hindgut (assessed by feces), as SARA is often associated with an increased amount of fermentable carbohydrates by-passing the rumen toward the hindgut, which could result in increased risk of hindgut acidosis, the development of diarrhea, and disturbance of hindgut bacteria [1, 9–11]. In previous studies, a grain-induced SARA challenge reduced the richness, evenness, and diversity of bacteria, and increased the abundance of nonstructural carbohydrates degraders (e.g., *Prevotella albensis*) in the feces [46, 47]. In the current study, no differences were observed between the groups in alpha diversity of fecal bacteria (i.e., observed ASV, Faith_pd, Shannon index, or evenness index) on d 21 postpartum. This may be linked with the potentially less harsh circumstances in the rumen by the gradual build-up of the supplemental compound feed (Table S1) in the beginning of lactation as compared with SARA-induction trials [8, 48]. In terms of beta diversity, principal-coordinate analysis of postpartum fecal bacterial communities did not allow distinguishing UN and SU cows (Fig. 2b). The limited bacterial shifts in the feces of SU and UN cows are in line with the lack of difference in fecal pH (6.47 vs. 6.41 of feces from SU and UN cows, respectively). Moreover, neither the postpartum fecal VFA concentration nor the DM content differed among groups. However, one family and six genera differed among the SARA groups on d 21 postpartum (Table 4): the relative abundance of the genus *Ruminococcus* was lower in the fecal bacterial communities of UN cows, while *Prevotellacea_UCG-001* increased in feces of SU cows. These genera, known to contain amylolytic bacteria [48–50], were also more abundant in the fecal bacterial community of cows fed high-starch challenged diets [51, 52]. Similar to starch-degrading bacteria, the relative abundance of the lactate-producing *Streptococcus* from the family Streptococcaceae increased in fecal samples of SU compared with UN cows, which was also observed in fecal samples of cows after a high-grain SARA challenge [53]. Nevertheless, pathogenic taxa such as *Escherichia coli* were not detected in the fecal samples of the current trial. This may be another illustration of the less harsh SARA conditions in the current trial compared with experimental, grain-induced SARA or post-ruminal infusion of easily fermentable carbohydrates to induce hindgut acidosis [52]. In these trials, the decreased hindgut/fecal pH as observed in SARA cows compared with non-SARA cows [8, 48, 54] could have enhanced proliferation of pathogenic taxa, especially *Escherichia* as the most common fecal pathogen [46, 55–57]. Thus, these results indicated that variation in reticular pH during a gradual build-up of the compound feed during a 3-week postpartum period was not associated with differences in fecal pH and VFA concentration, while some differences in the elative abundance of fecal genera were observed.

Odd- and branched-chain fatty acids have been identified as potential biomarkers in the rumen to reflect rumen function and to quantify the relative abundance of specific bacteria [57], because their synthesis is largely determined by fatty acid synthetases of the different micro-organisms [58–60]. As OBCFA in milk originate from rumen OBCFA, OBCFA in milk have been included in milk FA–based models to predict SARA [15, 16, 61]. Similarly to the use of milk OBCFA as biomarker for SARA diagnosis, Xin et al. [14] targeted fecal OBCFA (i.e., anteiso-C15:0, iso-C16:0, iso-C17:0, iso-C18:0, and total even-chain BCFA) to differentiate diarrheic and healthy calves. As SARA in dairy cows often is associated with diarrhea, we hypothesized fecal OBCFA could also be used to differentiate cows with or without SARA. In the current study, only iso-C15:0 and iso-C16:0 were higher or tended to be higher in UN compared with SU cows, which might be linked with more fermentable carbohydrates by-passing the rumen toward the hindgut of SU cows. Indeed, cellulolytic bacteria are characterized by relatively higher proportions of iso-FA while amylolytic bacteria are characterized by a relatively lower level of BCFA [62, 63]. However, these differences are limited, and some of the stronger SARA predictors in previous studies (e.g., iso-C14:0 [15], iso-C13:0 [62], and linear odd-chain FA such as C15:0 [15, 61]) did not differ in the feces of SU and UN cows. This coincides with the lack of difference in fecal DM content, indicating the absence of diarhea, which is often used as an on-farm indicator of SARA [1, 9], and is another indication of the relatively mild SARA conditions in the current animal cohort. In conclusion, only a minor reduction was observed in iso-fatty acid proportions in fecal fatty acids, particularly iso-C15:0 and iso-C16:0, of SARA SU cows compared with SARA UN cows.

Earlier work by our group indicated long-term persistence (> 1 year) of inter-animal differences in SARA susceptibility observed during early lactation [15]. If this persistence is the result of individual, animal-related characteristics, we hypothesized that SARA-susceptible cows could be distinguished from SARA unsusceptible cows during the prepartum period, prior to the postpartum dietary challenge. Despite a lack of difference between groups in fecal pH, some bacterial changes have been observed not only in the postpartum period, but also in the prepartum period. For beta diversity, PCoA based on Bray–Curtis distances of the prepartum fecal bacterial communities indicated a difference between UN and MO cows (Fig. 2). Although SU and UN cows did not show an overall distinct pattern of the fecal bacterial community, some genera differed (Table 5). For example, there was a lower relative abundance of *Ruminococcus* in the UN compared with the SU and MO cows (prepartum) and in the UN compared with the SU, MS, and MU cows (postpartum), which is consistent across the prepartum and postpartum periods. The genus *Ruminococcus* has often been reported to include starch-degrading bacteria [63]. Potentially, some starch-degrading bacteria, especially from the genus *Ruminococcus*, could be used as a prepartum indicator of postpartum SARA susceptibility. Further investigation is required to confirm this. In contrast, the genus *Lachnospiraceae_UCG-001* was more abundant in UN than SU cows in the prepartum period which was not reflected in postpartum period. The family Lachnospiraceae was found to be associated with the maintenance of gut health and to play a role as fiber-degrading bacteria [64]. Consistent with the bacterial changes postpartum, *Ruminococcus* was already enriched in the prepartum fecal bacterial communities of SARA SU cows, whereas *Lachnospiraceae_UCG-001* was decreased in these feces samples. Although our study indicated some bacterial differences both pre- as well as postpartum, the single-time-point sampling 1 week prior to calving and 3 weeks after calving is a limitation of our study. Nevertheless, we relied on studies by Grimm et al. [13] in which samples were taken 10 and 20 d after a dietary shift (hay or hay/barley diet) in horses and by Huang et al. [65] who sampled over three consecutive days from dairy cows. No major differences between samples taken at 10 and 20 d were observed by Grimm et al. [13], while Huang et al. [65] did not observe differences in diversity or relative abundances of fecal bacteria at the phylum and genus levels over the three consecutive days of sampling. As this was confirmed in earlier studies by Sadet-Bourgeteau et al. [66] and Blackmore et al. [67], it was concluded that the hindgut microbial ecosystem was established within 10 d after a dietary transition and that it remained stable within the same diet. Hence, in contrast to e.g., digestibility measurements which require the determination of absolute concentrations of a digestive marker, single-time point sampling might be reliable for microbial community analysis when considering relative abundances. Still, the added value of multiple samplings across and within days (diurnal variation) could be of interest when further finetuning the use of fecal bacterial indicators for disease identification in the future.

As some differences were observed within the prepartum fecal bacterial community of cows with distinct postpartum SARA susceptibility, we hypothesized that similarity in the prepartum bacterial community (i.e., Bray–Curtis distance) can be used to cluster cows with different postpartum SARA susceptibility. Despite the major differences in bacterial composition between the prepartum and postpartum periods, the prepartum fecal bacterial communities of 29 cows were distributed into two main clusters that are, to some extent, in agreement with the SARA susceptibility groups. Cluster 1 contained mainly SU cows, whereas cluster 2 contained mainly UN and MO cows, suggesting prepartum Bray–Curtis similarity could potentially be used to roughly identify SU cows. The fecal bacterial community is shaped by diet, environmental changes, and the host (genetic effects) [21]. In particular, host (genetic) effects may determine the hindgut bacterial community, which may be associated with the animal’s health status [20, 21] and may be resilient to dietary perturbations [22]. Conversely, we speculate that the hindgut bacterial community structure of SU cows may be less resilient to the prepartum to postpartum dietary shift. Although the fecal bacterial community differed considerably between the prepartum and postpartum periods, the shift in the bacterial community from the prepartum to postpartum periods was independent of differences between the cows regarding SARA susceptibility. This suggests that the dietary shift from prepartum to postpartum is the primary effect influencing the bacterial structure. This is in line with a previous observation by Mohammed et al. [68], albeit in the rumen bacterial community, where postpartum shifts in the bacterial community appear to be independent of the differences in the severity of SARA postpartum. Taken together, differences in prepartum fecal bacterial communities could already alert risks of postpartum SARA development. Nevertheless, shifts in the bacterial community from the prepartum to postpartum periods was independent of differences between the cows regarding SARA susceptibility.

## Conclusions

Variation in reticular pH during a 3-week postpartum period was not associated with differences in fecal pH and VFA concentration. Nevertheless, the copy number of fecal bacteria and methanogens of UN cows was higher than MS/SU cows in the postpartum period, while the genera *Ruminococcus* and *Prevotellacea_UCG-001* were proportionally less abundant in UN compared with SU cows. This change was accompanied by a minor reduction in iso-BCFA proportions in fecal fatty acids of SU cows, particularly iso-C15:0 and iso-C16:0 (trend). In the prepartum period, the relative abundance of *Ruminococcus* was decreased in the feces of UN cows, whereas *Lachnospiraceae_UCG-001* was increased. Nevertheless, no differences were observed in fecal VFA or OBCFA profiles prepartum. Prepartum Bray–Curtis similarity could potentially give a first indication of postpartum SARA susceptibility. As such, differences in prepartum fecal bacterial communities potentially could already alert for SARA postpartum development. Our results generated knowledge on the association between fecal bacteria and SARA development which could be further explored in a prevention strategy.

## Supplementary Information

**Additional file 1: Table S1.** Linear build-up of the supplemental part of the diet, individually supplied to the cows during milking and via the concentrate dispenser (kg/d).^1^Contains (g/kg product): dry beet pulp (100), soybean meal (270), wheat (85), maize (430), molasses (70), salt (6), feed phosphate (10), micro minerals (10), lignin-sulfonate (10), chalk (4), and magnesium oxide (5).^2^Contains (g/kg product): beet pulp (370), soybean meal (210), wheat (185), maize (120), molasses (50), salt (12), soy oil (10), feed phosphate (10), micro minerals (10), lignin-sulfonate (10), chalk (8), and magnesium oxide (5).^3^Covasoy = formaldehyde-treated soybean meal to bypass rumen degradation.^4^Supplement start was given from day 3 after calving. **Table S2.** Primers used to quantify selected ruminal microbial groups using a real-time quantitative polymerase chain reaction assay. ^1^F = forward; R = reverse. **Table S3.** Median and mean diurnal time of pH below 6 (min/d) in the 3-week postpartum period and 1-week prepartum period based on real calving dates in relation to variation in SARA susceptibility over the first 3 postpartum weeks

## Data Availability

Sequence files associated with each sample have been submitted to the NCBI Sequence Read Archive (SRA; https://www.ncbi.nlm.nih.gov/sra; Accession Number: PRJNA774499).

## Abbreviations

VFA: Volatile fatty acids
OBCFA: Odd- and branched-chain fatty acids
SU: Susceptible group
MS: Moderately susceptible group
MU: Moderately unsusceptible group
UN: Unsusceptible group
MO: Moderate group
SARA: Subacute ruminal acidosis
ILVO: Institute for Agriculture, Fisheries and Food
rRNA: Ribosomal RNA
QIIME2: Quantitative Insights Into Microbial Ecology 2
ASV: Amplicon sequence variants
qPCR: Quantitative polymerase chain reaction
ANOVA: One-way analysis of variance
PCoA: Principal-coordinate analysis
PERMANOVA: Permutational multivariate analysis of variance
ANCOM: Analysis of composition of microbiomes
DMI: Dry matter intake
Lac3 and Lac20: D 3 and 20 in lactation
SEM: Standard error of the mean.
*P*_adj_: *P* value adjusted for false discovery rate at 5%
