# Supplementary material for: Susceptibility of dairy cows to subacute ruminal acidosis is reflected in both prepartum and postpartum bacteria as well as odd- and branched-chain fatty acids in feces
Source: J Anim Sci Biotechnol. 2022 Oct 5;13:87. doi: 10.1186/s40104-022-00738-8 (PMC9533591; doi:10.1186/s40104-022-00738-8)
Supplement: Supplementary file 1 — Additional file 1: Table S1. Linear build-up of the supplemental part of the diet, individually supplied to the cows during milking and via the concentrate dispenser (kg/d).1Contains (g/kg product): dry beet pulp (100), soybean meal (270), wheat (85), maize (430), molasses (70), salt (6), feed phosphate (10), micro minerals (10), lignin-sulfonate (10), chalk (4), and magnesium oxide (5).2Contains (g/kg product): beet pulp (370), soybean meal (210), wheat (185), maize (120), molasses (50), salt (12), soy oil (10), feed phosphate (10), micro minerals (10), lignin-sulfonate (10), chalk (8), and magnesium oxide (5).3Covasoy = formaldehyde-treated soybean meal to bypass rumen degradation.4Supplement start was given from day 3 after calving. Table S2. Primers used to quantify selected ruminal microbial groups using a real-time quantitative polymerase chain reaction assay. 1F = forward; R = reverse. Table S3. Median and mean diurnal time of pH below 6 (min/d) in the 3-week postpartum period and 1-week prepartum period based on real calving dates in relation to variation in SARA susceptibility over the first 3 postpartum weeks [file 40104_2022_738_MOESM1_ESM.docx]

**Additional file 1**

| Item | Supplement start amount^4^ | Build-up duration, d | Supplement 20 d amount |
| --- | --- | --- | --- |
| Balanced compound feed A^1^ | 0.2 | 20 | 2 |
| Balanced compound feed B^2^ | 1.5 | 10 | 4 |
| Covasoy^3^ | 0.2 | 7 | 1 |
| Soybean meal | 0.3 | 20 | 0.3 |

Table S1. Linear build-up of the supplemental part of the diet, individually supplied to the cows during milking and via the concentrate dispenser, kg/d

^1^Contains (g/kg product): dry beet pulp (100), soybean meal (270), wheat (85), maize (430), molasses (70), salt (6), feed phosphate (10), micro minerals (10), lignin-sulfonate (10), chalk (4), and magnesium oxide (5)

^2^Contains (g/kg product): beet pulp (370), soybean meal (210), wheat (185), maize (120), molasses (50), salt (12), soy oil (10), feed phosphate (10), micro minerals (10), lignin-sulfonate (10), chalk (8), and magnesium oxide (5)

^3^Covasoy = formaldehyde-treated soybean meal to bypass rumen degradation

^4^Supplement start was given from day 3 after calving

| Target group | Oligo name^1^ | Primer Sequence (5’→3’) | Amplicon length, bp | Reference |
| --- | --- | --- | --- | --- |
| General bacteria | Gbacteria_F | CGGCAACGAGCGCAACCC | 130 | [38] |
|  | Gbacteria_R | CCATTGTAGCACGTGTGTAGCC |  |  |
| Anaerobic fungi (*Neocallimastigales*) | Neo QPCR F | TTGACAATGGATCTCTTGGTTCTC | 110 | [39] |
|  | Neo QPCR R | GTGCAATATGCGTTCGAAGATT |  |  |
| Methanogens | qmcrA F | TTCGGTGGATCDCARAGRGC | 140 | [41] |
|  | qmcrA R | GBARGTCGWAWCCGTAGAATCC |  |  |
| Protozoa | 316F | GCTTTCGWTGGTAGTGTATT | 223 | [40] |
|  | 539R | CTTGCCCTCYAATCGTWCT |  |  |

Table S2. Primers used to quantify selected ruminal microbial groups using a real-time quantitative polymerase chain reaction assay

^1^F = forward; R = reverse

Table S3. Median and mean diurnal time of pH below 6 (min/d) in the 3-weeks postpartum period and 1-week prepartum period based on real calving dates in relation to variation in SARA susceptibility over the first 3 postpartum weeks

| Item | Groups | | | | | | | | | | |
| --- | --- | --- | --- | --- | --- | --- | --- | --- | --- | --- | --- |
| **Prepartum** |  | | | | | | | | | | |
|  | Susceptible group | | | | | | | | | | |
| Cow | SU1 | SU2 | SU3 | SU4 | SU5 | SU6 | SU7 | SU8 | SU9 | SU10 |  |
| Median time of pH below 6, min/d | 704 | 0.00 | 284 | 410 | 0.00 | 246 | 0.00 | 0.00 | 0.00 | 252 |  |
| Mean time of pH below 6, min/d | 710 | 0.00 | 270 | 310 | 0.00 | 270 | 0.00 | 0.00 | 0.00 | 260 |  |
| Days with time of pH below 6 > 330 min/d in 7 d | 5 | 0 | 1 | 1 | 0 | 1 | 0 | 0 | 0 | 1 |  |
|  | Moderate group | | | | | | | | | | |
| Cow | MS-2 | MS-3 | MS-5 | MS-6 | MU-1 | MU-2 | MU-3 | MU-4 | MU-6 | MU-11 |  |
| Median time of pH below 6, min/d | 20.0 | 0.00 | 0.00 | 0.00 | 30.0 | 0.00 | 40.0 | 0.00 | 0.00 | 10.0 |  |
| Mean time of pH below 6, min/d | 0.00 | 0.00 | 0.00 | 0.00 | 0.00 | 0.00 | 0.00 | 0.00 | 0.00 | 0.00 |  |
| Days with time of pH below 6 > 330 min/d in 7 d | 0 | 0 | 0 | 0 | 0 | 0 | 0 | 0 | 0 | 0 |  |
|  | Unsusceptible group | | | | | | | | | | |
| Cow | UN1 | UN2 | UN3 | UN4 | UN5 | UN6 | UN7 | UN8 | UN9 | ⸻ |  |
| Median time of pH below 6, min/d | 0.00 | 0.00 | 0.00 | 0.00 | 0.00 | 0.00 | 0.00 | 0.00 | 0.00 | ⸻ |  |
| Mean time of pH below 6, min/d | 0.00 | 0.00 | 0.00 | 0.00 | 0.00 | 0.00 | 0.00 | 0.00 | 0.00 | ⸻ |  |
| Days with time of pH below 6 > 330 min/d in 7 d | 0 | 0 | 0 | 0 | 0 | 0 | 0 | 0 | 0 | ⸻ |  |
| **Postpartum** |  | | | | | | | | | | |
|  | Susceptible group | | | | | | | | | | |
| Cow | SU1 | SU2 | SU3 | SU4 | SU5 | SU6 | SU7 | SU8 | SU9 | SU10 | ⸻ |
| Median time of pH below 6, min/d | 660 | 540 | 480 | 330 | 315 | 230 | 195 | 180 | 150 | 130 | ⸻ |
| Mean time of pH below 6, min/d | 624 | 446 | 573 | 309 | 476 | 253 | 272 | 177 | 222 | 203 | ⸻ |
| Days with time of pH below 6 > 330 min/d in 21 d | 18 | 14 | 16 | 10 | 10 | 4 | 7 | 2 | 5 | 2 |  |
|  | Moderately susceptible group | | | | | | | | | | |
| Cow | MS-1 | MS-2 | MS-3 | MS-4 | MS-5 | MS-6 | MS-7 | ⸻ | ⸻ | ⸻ | ⸻ |
| Median time of pH below 6, min/d | 105 | 80.0 | 60.0 | 60.0 | 10.0 | 10.0 | 0.00 | ⸻ | ⸻ | ⸻ | ⸻ |
| Mean time of pH below 6, min/d | 126 | 147 | 78.6 | 74.3 | 143 | 72.9 | 83.8 | ⸻ | ⸻ | ⸻ | ⸻ |
| Days with time of pH below 6 > 330 min/d in 21 d | 1 | 2 | 0 | 0 | 2 | 4 | 2 | ⸻ | ⸻ | ⸻ | ⸻ |
|  | Moderately unsusceptible group | | | | | | | | | | |
| Cow | MU-1 | MU-2 | MU-3 | MU-4 | MU-5 | MU-6 | MU-7 | MU-8 | MU-9 | MU-10 | MU-11 |
| Median time of pH below 6, min/d | 30.0 | 30.0 | 20.0 | 15.0 | 15.0 | 0.00 | 0.00 | 0.00 | 0.00 | 0.00 | 0.00 |
| Mean time of pH below 6, min/d | 44.8 | 42.9 | 55.2 | 53.6 | 36.4 | 33.6 | 22.9 | 21.4 | 17.1 | 15.2 | 13.8 |
| Days with time of pH below 6 > 330 min/d in 21 d | 0 | 0 | 1 | 0 | 0 | 0 | 0 | 0 | 0 | 0 | 0 |
|  | Unsusceptible group | | | | | | | | | | |
| Cow | UN1 | UN2 | UN3 | UN4 | UN5 | UN6 | UN7 | UN8 | UN9 | UN10 | ⸻ |
| Median time of pH below 6, min/d | 0.00 | 0.00 | 0.00 | 0.00 | 0.00 | 0.00 | 0.00 | 0.00 | 0.00 | 0.00 | ⸻ |
| Mean time of pH below 6, min/d | 10.0 | 5.71 | 5.24 | 3.33 | 3.00 | 1.43 | 0.00 | 0.00 | 0.00 | 0.00 | ⸻ |
| Days with time of pH below 6 > 330 min/d in 21 d | 0 | 0 | 0 | 0 | 0 | 0 | 0 | 0 | 0 | 0 | ⸻ |
